# Retraction Note: MicroRNA-506 inhibits tumor growth and metastasis in nasopharyngeal carcinoma through the inactivation of the Wnt/β-catenin signaling pathway by downregulating LHX2

**DOI:** 10.1186/s13046-021-02113-3

**Published:** 2021-09-30

**Authors:** Tian-Song Liang, Ying-Juan Zheng, Juan Wang, Jing-Yi Zhao, Dao-Ke Yang, Zhang-Suo Liu

**Affiliations:** Department of Radiotherapy, the First Affiliated Hospital of Zhengzhou University, Zhengdong Branch, Zhengzhou, 475000 Henan Province People’s Republic of China

**Retraction Note: J Exp Clin Cancer Res 38, 97 (2019)**

**https://doi.org/10.1186/s13046-019-1023-4**

The Editor-in-Chief has retracted this article. Concerns have been raised regarding a number of figures, specifically:

- Figure 9A: the panels for 0 h/siRNA-LHX2 and 0 h/miR-506 inhibitor + siRNA-LHX2 appear to partially overlap
- Figure 9B: the panels for 0 h/Blank and 0 h/NC appear to partially overlap
- Figure 9B: the panels for 0 h/miR-506 mimic and 0 h/miR-506 inhibitor appear to be identical

Additionally, the background of the western blots in Figures 2B, 4A, 5B, 5D, 5H and 5 J appear to look unexpectedly clean. Author Zhang-Suo Liu has also stated that he was unaware of the submission and publication of this article. The Editor-in-Chief therefore no longer has confidence in the reliability of the data reported in the article.

The authors have not responded to correspondence regarding this retraction notice.

